# The Endogenous Pain Modulatory System as a Healing Mechanism: A Proposal on How to Measure and Modulate It

**DOI:** 10.3390/neurosci5030018

**Published:** 2024-07-07

**Authors:** Paulo S. de Melo, Kevin Pacheco-Barrios, Anna Marduy, Karen Vasquez-Avila, Marcel Simis, Marta Imamura, Alejandra Cardenas-Rojas, Alba Navarro-Flores, Linamara Batistella, Felipe Fregni

**Affiliations:** 1 Neuromodulation Center and Center for Clinical Research Learning, Spaulding Rehabilitation Hospital and Massachusetts General Hospital, Harvard Medical School, 96 13th Street, Charlestown, Boston, MA 02141, USA; 2 Unidad de Investigación para la Generación y Síntesis de Evidencias en Salud, Universidad San Ignacio de Loyola, Lima 15024, Peru; 3 Hospital das Clinicas HCFMUSP, Faculdade de Medicina, Universidade de São Paulo, São Paulo 05508-060, Brazil; 4 Escuela de Medicina, Universidad Cesar Vallejo, Trujillo 13001, Peru

**Keywords:** biomarkers, cortical excitability, chronic pain, healing, neuroscience, transcranial magnetic stimulation

## Abstract

Background: Chronic pain is highly burdening and multifactorial in etiology. The endogenous-pain-healing system restores body tissue to a non-painful state after an injury leading to pain, and its disruption could represent a relevant mechanism, especially for nursing interventions. Aim: To review the literature and summarize the results that support this hypothesis. Methods: We hypothesized that the mechanism behind this system mainly depends on the endogenous pain modulatory system (EPMS), which is responsible for inhibiting pain after tissue healing is complete and facilitating it when tissue damage is still present. Different biomarkers can quantify EPMS functioning. We reviewed the literature and included relevant information regarding this hypothesis. Results: First, conditioned pain modulation (CPM) measures pain inhibition and is a possible predictor for pain chronification. Second, motor cortex excitability measures the cortical control of the EPMS, which can be assessed through transcranial magnetic stimulation (using intracortical inhibition) or electroencephalography. Modifiable factors disrupt its functioning, such as sleep deprivation, medication overuse, and mental health status, but could be protective, such as exercise, certain medications, mind–body techniques, and non-invasive neuromodulation therapies. The acquisition of neurophysiological knowledge of how the chronicity of pain occurs and the EPMS involvement in this process may allow for better management of these patients. Conclusions: We raised the hypothesis that the impairment of the EPMS (altered cortical excitability and descendent pain modulation pathways) seems to be related to the disruption of the pain healing process and its chronicity. Further longitudinal studies evaluating the relationship between these biomarkers and chronic pain development are necessary.

## 1. Introduction

Chronic pain is a leading cause of years lost disability [1]. Its prevalence varies between 11 and 40% [2]. The Institute of Medicine estimated that chronic pain affects one-third of Americans, costing around USD 600 billion per year in health care costs and lost productivity [3].

Although chronic pain does not affect all people equally, biopsychosocial processes predispose the development and persistence of chronic pain, like depression, anxiety, post-traumatic stress, poor coping skills, catastrophization, low educational attainment, cultural beliefs, poor social support, genetics, age, sex, sleep, hormones, and endogenous opiate systems [4,5,6].

Several studies have found alterations in the central nervous system related to chronic pain [7,8,9,10], and several biomarkers are being studied for measuring the healing process after injury and predicting pain chronification [11]. Amongst them, the endogenous pain modulation system seems to be an important factor in perpetuating pain in chronic pain populations [12,13]. However, how these factors influence the development of the disease is not well established.

This manuscript will suggest a model in which the endogenous pain modulation system (EPMS) is part of a brain-based pain healing mechanism from an initial injury or tissue damage, which is disrupted among those with chronic pain. We propose that biomarkers of the EPMS, such as conditioned pain modulation and cortical excitability, could represent the healing mechanism and could be validated for clinical use and personalized pain management.

## 2. The Definition of Healing

The definition of healing is broad and different depending on the source. The current literature has defined healing as (1) the restoration of tissue architecture and function following an injury; (2) a mechanism to maintain the integrity and function of the body in response to acute and chronic injuries and disease states; (3) a sequence of complex events to repair damage, prevent infection, and restore the function of tissue after injury or insult; and (4) a natural and continuous process in any living organism with of two essential components: regeneration and repair. For the scope of this paper, we defined healing as the process of restoring health (function and integrity) from an unbalanced state or damaged organism.

Healing a tissue occurs in phases such as inflammation, proliferation, angiogenesis, restoration, and remodeling [14]. The peripheral inflammation process plays an important role in healing and pain transmission. For instance, neutrophins, like nerve growth factor (NGF) and brain-derived neurotrophic factor (BDNF), are key neuronal regulators on the periphery, possibly mediating the transition from acute to chronic pain [15,16,17,18,19,20]. NGF regulates sensory and sympathetic neuron survival, growth, proliferation, and plasticity; also, it is a critical player in wound healing, regulating the survival of non-neuronal cells in the inflammation and repair process [17]. Additionally, BDNF is one of the mediators of the central sensitization of pain in the spinal cord, being localized in injured tissues at the beginning of the inflammatory process [21,22].

Pain is an intrinsic part of the healing process, and it is defined as an unpleasant sensory and emotional experience associated with actual or potential tissue damage [23]. It is hypothesized that organisms experience pain through the central nervous system as an attempt to protect damaged tissue (inducing aversive behaviors), being the first step in the healing mechanism: the interruption of the injury. This characterizes the pain as a self-limited process—once the source of injury is stopped, the danger for the tissue is not present, and the brain does not have to be protective—producing pain—anymore [24]. Therefore, once the acute danger period is over, the pain is no longer necessary but a disease itself [25]. Moreover, there is no specific threshold for when acute pain becomes chronic; it is accepted that pain persisting beyond the expected healing period (generally 3 months) is pathological.

## 3. EPMS as a Key Component of the Healing Process

**Figure 1:**
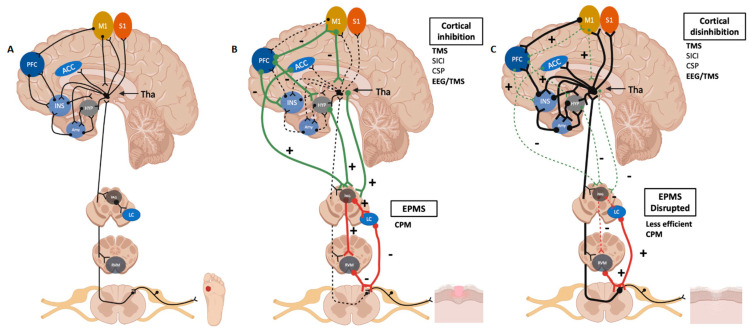
We can observe the endogenous pain modulation pathways. Regions highlighted in our schematic figure are M1, primary motor cortex; S1, primary somatosensory cortex; PFC, prefrontal cortex; ACC, anterior cingulate cortex; INS, insula; Hyp, hypothalamus; amy, amygdala; Tha, thalamus; PAG, periaqueductal gray matter; LC, locus coeruleus; RVM, rostroventral medulla. (**A**) The physiologic afferent pathways of pain sensation and perception are represented. (**B**) The role of the highlighted regions in pain modulation during the healing process. There is a feedback loop between ongoing inflammation associated with the healing process and the activation of the EPMS to mitigate pain after complete healing. (**C**) Pain facilitation when the endogenous pain modulation system is disrupted, such as in chronic pain states. Created with BioRender.com.

The EPMS is a major contributor to pain inhibition in situations where there is no more danger and to facilitation in situations where danger to the tissue is still present. In general, neuroplasticity associated with pain modulation follows classic mechanisms: long-term potentiation (LTP) and long-term depression (LTD). Both mechanisms have been demonstrated to be related to pain modulation, depending on the specific neuron function in the EPMS [26,27]. Although these mechanisms could occur in several levels within the pain pathways (peripheral, spinal, and supraspinal), there are two main levels where endogenous mechanisms may modulate pain perception: (1) dorsal inhibitory interneurons that utilize GABA, glycine, or opioid receptors and provide feed-forward or feedback inhibition to the spinothalamic and spinobulbar pathways; and (2) descending pathways originated in cortical regions (prefrontal and sensorimotor cortices) and the brainstem, which inhibit or facilitate peripheral nociceptive input on the dorsal horn inhibitory interneurons. In this process, the midbrain periaqueductal gray (PAG) and the rostroventral medulla (RVM) are key structures—we call these pathways the EPMS (Figure 1).

Therefore, in healthy conditions, the EPMS seems to control pain during the healing process based on constant peripheral input (inflammatory and anti-inflammatory signals) and does not allow the perpetuation of the symptom more than necessary for tissue protection (Figure 1B). We hypothesize there is a neuro-immune crosstalk between the ongoing levels of inflammatory mediators and growth factors released by immune cells [28] according to the healing stage. These signals will activate the EPMS in a bottom-up manner (via spinal tracts), forming a feedback loop that controls ongoing pain during and after healing. We hypothesize the neuro-immune crosstalk is mediated by peripheral nerves (local inflammation) and the vagus nerve (systemic and visceral inflammation). The inefficient control of pain during the healing process may lead to the chronicity of the symptom longer than the time taken to heal tissue damage. In this scenario, due to multiple causes affecting the effective activation of the EPMS, even though the tissue is partially or fully recovered, the pain persists, leading to unpleasant sensory, emotional, and cognitive experiences.

If not treated correctly, these patients may never recover the function of the damaged tissue and overall wellbeing. The reason for healing mechanism disruption is still unclear, but it may require multifactorial changes in pain main aspects: nociception (signal transduction component), pain perception (sensory–discriminatory component), suffering (emotional-affective component), and pain behaviors (cognitive–behavioral–motivational component). Therefore, the nociception component, solved by inflammation and tissue healing mechanisms, is the “tip of the iceberg” for the entire process [28]. The persistence of pain may be related to an aberrant course of endogenous pain modulation naturally present in the healing process in healthy individuals.

Dysfunctional EPMS is a potential mechanism in chronic pain syndromes related to central sensitization, in which there are functional changes in the pain pathways that result in a shift from “high threshold nociception to low-threshold hypersensitivity” and brain maladaptive changes [29,30,31].

Therefore, a current challenge in the pain field is to develop accurate measurements of pain chronicity. We hypothesize that the EPMS (including supraspinal and spinal mechanisms) controls pain immediately after an injury as an intrinsic factor in the healing process, which prevents the development of chronic pain in the future in healthy populations. A way to analyze this phenomenon is to measure endogenous pain modulation system efficiency through CPM (dynamic quantitative sensory testing) and cortical excitability (indexed by transcranial magnetic stimulation (TMS) or electroencephalogram (EEG)) (Figure 1C).

### 3.1. Measuring the EPMS: Conditioned Pain Modulation (CPM)

Studies suggest that the EPMS is less efficient in chronic pain conditions [32,33]. This may explain one of the reasons for the perpetuation of pain longer than usual and even after the time it takes to heal the initial injury. One of the mechanisms developed to measure EPMS efficiency is CPM [32,33,34,35].

CPM is a measure that quantifies the effectiveness of the pain inhibitory and facilitatory pathways. It has become a “surrogate measure of the brain’s capacity for activating endogenous analgesia” [36]. The CPM paradigm relies on the belief that “pain inhibits pain”; for example, after the application of a nociceptive stimulus, the application of a second different nociceptive stimulus reduces the pain generated from the first stimulus. This phenomenon is considered to occur due to the modulation effect between the descending pain pathways.

Calculating CPM is performed using a dynamic quantitative sensory testing (QST) technique, in which central pain pathways are evaluated. Using the example previously mentioned, the first stimulus would be considered the test stimulus (TS), and the second stimulus would be called the conditioning stimulus (CS) [37]. Nonetheless, this is where all similarities regarding CPM protocols end.

At present, there is no standardized CPM protocol [38]. Different types of stimulus and outcome measures are used in practice. For conditioned stimulus, the most common technique used is cold pressor pain (CPP)—cold water immersion of the upper limb—but other techniques can be used as well, such as a noxious heat stimulation or the ischemic arm technique. In contrast, for test stimuli, thermal, mechanical, electrical, chemical, or laser are used [36].

However, there may appear to be an agreement among scientists to perform CPM in steps: applying the second nociceptive stimulus after the first—not simultaneously [36]. When the initial test stimulus score decreases after the intervention, reflected in a positive CPM, the endogenous pain modulation system is considered to be preserved due to its efficacy in inhibiting pain [38].

An abnormal CPM paradigm has been widely associated with many chronic pain conditions [36] as well as with the risk of the development of post-surgical chronic pain [38]. However, a causal relationship has not been established yet. Yarnitsky et al. suggested using the CPM paradigm as a tool to categorize patients by pain modulation profiles [39]. Patients with an inefficient CPM—increased facilitation pain pathways and decreased inhibitory pain pathways—are more likely to present a pro-nociceptive profile, which could eventually lead to a low pain threshold and a higher risk of chronic pain in the future, whereas patients with an efficient CPM would fall into the anti-nociceptive profile and present a higher pain threshold [39].

Chronic pain development in different conditions involves high inter-patient variability, thus being difficult to manage and resolve in the healing process. This high inter-variability can be depicted through endogenous pain modulation paradigms such as CPM. Several studies have conveyed CPM as a malleable and remarkably neuroplastic paradigm, as it can worsen or improve according to pain aggravation or alleviation, respectively.

The EPMS can be characterized by three different mechanisms in which CPM might be involved: spinal, descending inhibitory controls in the subcortical regions, and cortical mechanisms [40,41]. Moreover, several studies have identified CPM modulation through neurotransmitters such as noradrenaline and serotonin, indicating that medications that affect the uptake of these neurochemical pathways can alter CPM efficiency [42]. This contributes to the theory that deficient CPM can be “normalized” with specific treatments that aim at treating persistent pain. For instance, studies have conveyed that joint replacement surgery has led to the normalization of CPM paradigms in individuals with osteoarthritis [43,44].

CPM has been reported as a predictor of chronic pain development and treatment response according to its efficiency in individuals at baseline. A study investigating CPM performance in individuals before a thoracotomy showed that those with less efficient CPM have a higher chance of developing chronic or persistent pain compared with those with a regular functioning CPM. Moreover, Yarnitsky et al. have shown that medication targeting the descending pain modulation system seems to be effective only in individuals with malfunctioning CPM compared with those with high-functioning CPM [39,45]. Furthermore, along with pain alleviation, regular levels of CPM were restored. This relates to another study in which individuals with less effective CPM needed more morphine to treat their pain when compared with those with normal functioning CPM [46]. Considering CPM as a method of measuring the EPMS, we can relate its predictor characteristic as a marker for healing. Thus, when an individual’s CPM is inefficient, it can be related to increased chronic pain susceptibility, therefore disrupting the healing process. Additionally, pain alleviation can lead to restoring CPM to its regular levels and, consequently, resume healing.

These findings suggest that CPM could predict treatment response and chronic pain prognosis and reflect the operation of the descending pain inhibitory pathways. Hence, if we assume the chronification of pain as the disruption of healing, we could accept CPM as a marker of efficient healing, thus using it to promote individualized treatments for those with deficient endogenous pain modulation systems. It is worth mentioning that sometimes, changes in CPM will not occur, and CPM efficiency and pain severity may not always be linked to disease severity [34,47]. Thus, CPM may only be a marker for healing for specific populations in which CPM is deficient.

### 3.2. A Critical Modulator of the Endogenous Pain Inhibitory System: The Primary Motor Cortex

Another hypothesized factor as an endogenous modulator of pain is motor cortex excitability. It is measured by single or pair-pulsed TMS (TMS). The use of paired-pulse TMS is a way to measure intracortical inhibition by the suppression of motor evoked potentials with interstimulus intervals between 1 and 5 ms due to the activation of GABAergic inhibitory interneurons [48].

Reorganization of the primary motor cortex (M1) has been detected in several chronic pain conditions. This phenomenon is thought to be initiated by changes in intracortical excitability, with a reduction in GABA-ergic inhibition and an enhancement of glutamatergic facilitation, leading to increased excitation and central sensitization [49].

However, studies have shown that pain relief in chronic pain populations is related to an increase in cortical excitability after anodal stimulation, meaning that the facilitation of the motor cortex can also be related to less pain in these populations [50].

We can explain the role of M1 in pain perception by two mechanisms: (1) the control of the descending pain modulation pathway (measured by CPM), supported by a recent meta-analysis showing that M1 stimulation produces an increase in CPM efficacy [51], through intrinsically related brain areas connected with M1 (such as periaqueductal gray matter, pregenual anterior cingular cortex, and medial prefrontal cortex [52,53]); and (2) the control of the thalamic processing, since the pain inputs from the primary motor cortex and areas associated with the pain neuromatrix overlap, these areas receive peripheral inputs via thalamic relay nuclei, somatosensory cortex, premotor cortex, and sensory association areas (Figure 1A). This is supported by studies showing that stimulation of M1 induces corticothalamic inhibition of the ventral posterolateral nucleus (VPL). This area is responsible for discriminatory sensitivity, and the ventral posteromedial nucleus (VPM) is responsible for nociceptive sensation [54,55,56,57].

Therefore, higher motor cortex excitability in healthy individuals—or provided by neuromodulation interventions such as tDCS in those with chronic pain—may increase the activation of descending pain modulation pathways, which control pain sensation through inhibitory interneurons in the medulla dorsal horn [52].

Moreover, intracortical facilitation and inhibition mechanisms are shown to be mediated by GABAergic and glutamatergic pathways [58], suggesting that neurochemical imbalances can trigger healing disruptions, thus promoting chronic pain [59]. Additionally, compensatory mechanisms, including sensory deafferentation and immobilization, may be contributing predictors for the development of chronic pain, promoting the loss of GABA-mediated inhibition in motor cortical areas, further leading to a reduction in intracortical facilitation and inhibition. The mechanisms of cortical disinhibition suggest a disruption in the healing process provoked by compensatory mechanisms primed through the original injury [60].

The M1 excitability disruption in individuals with chronic pain and the evidence of its influence in the EPMS suggest that the facilitation and inhibition of M1 play an important role in the healing process, controlling pain perception after an injury. In our cohort study (the DEFINE study [61]), we performed a cross-sectional modeling analysis in 107 subjects with osteoarthritis to investigate the association of clinical and sociodemographic variables and cortical excitability. We have shown that subjects with higher radiographic disease severity (conveyed by the Kellgren–Lawrence classification) and low pain levels have higher intracortical motor cortex inhibition, thus showing that subjects with an appropriate level of compensation and activation of the cortical mechanism of EPMS (in this case the M1 inhibition) have less pain [62]. However, this was a cross-sectional study, and it was only possible to study the association between the variables. Noteworthy, when cortical excitability is disrupted, the pain modulation is compromised, and chronic pain may develop. Therefore, we hypothesized that the adequate balance among inhibition and facilitation of cortical excitability—with the descending endogenous pain modulation pathway—prevents chronic pain development as a healing mechanism. Although we focused on the M1 as one of the main cortical modulators of EPMS, the prefrontal cortex, cingulate cortex, and insula are areas to consider. More EEG and TMS studies are needed to explore inhibition–facilitation balance on these areas to understand its role on the EPMS control and healing process [63,64].

Previous research has found several factors that may contribute to the disruption or restoration of a balanced EPMS. Once the EPMS is confirmed as a main component and modulator of the healing mechanism, avoiding pain chronification, healthcare providers would be able to focus on bypassing the aspects that disrupt and incentivize all elements that enhance the pain modulation system balance. This approach may be the alternative in the prevention and treatment of chronic pain.

## 4. Factors That Disrupt the Balance of the Pain Healing Mechanisms

Previous studies have tried to identify predictors for CPM efficacy and predictors for chronification of pain. This endogenous pain modulation system can be disrupted at different levels, leading to the chronification of pain. Several studies have investigated different factors such as sleep, mood disorders, cognition, and medication overuse. However, the association between these factors and the chronification of pain is not clear yet.

### 4.1. Sleep Quality

Poor sleep quality and sleep disorders have been described in several chronic pain conditions; around 40% of people with chronic low back pain and chronic neck pain suffer from sleep deprivation [65]. Sleep deprivation has been shown to affect the endogenous modulatory pathway by decreasing inhibitory and enhancing facilitation pathways, including cortical excitability, neurotransmission, and neurogenesis [66]. Supporting this notion, in animal studies, sleep deprivation showed an increase in cortical excitability after 4 h, modulating cortical activity through possibly involving Ca^2+^ dependent mechanisms [67]. Similarly, recent research into healthy humans using single-pulse TMS has conveyed that sleep deprivation increases cortical excitability with a lower short intracortical inhibition [68,69]. Moreover, sleep deprivation increases the expression of glutamate receptors in the rat hippocampus. This is involved in increased susceptibility to short- and long-term depression in that area. Staffe et al., 2019 described a significant disruption of CPM and an increase in temporal summation in patients with sleep deprivation [70]. Therefore, sleep deprivation might contribute to the chronification of pain; however, there is a need for more studies to detangle their neurophysiological relationship and interventions.

### 4.2. Medication Overuse and Opioids

Medication overuse has been described as a possible factor contributing to pain chronification [71]. Animal studies have also shown disruption of the descending system by medications such as opioids and triptans, leading to the chronification of pain. Coppola et al., 2012 described how it contributes to headache chronification in 29 patients with chronic migraine and medication overuse [71]. They showed that NSAIDs and triptans overuse induce somatosensory evoked potential sensitization that persisted over time (lack of habituation), and both influence the excitability of the somatosensory and motor cortices. Moreover, Chen et al., 2017 showed an increase in gray matter volume of the thalamus in 27 patients with medication overuse compared with controls, similar to other chronic pain conditions [72]. Moreover, chronic use of opioids leads to opioid-induced hyperalgesia and a defective descending endogenous pain inhibitory system. We hypothesize that since one of the main effectors of EPMS are endogenous opioid peptides, the administration of constant exogenous opioids could downregulate the EPMS mechanism (similar to the downregulation of the hypophysis–adrenal axis by chronic administration of corticoids), inducing a lack of effective response. Recent studies investigated the effects of opioid infusions and, even in healthy individuals, most showed that opioid infusions lead to higher pain intensity, allodynia, and greater incidence of chronic pain [73,74,75,76]. These findings show a significant dysfunction in the EPMS in the chronic use of opioids.

### 4.3. Poor Mental Health

Chronification of pain has been related to emotional-related pathways by shifting from sensory regions in acute pain (lateral network) to emotional and limbic structures (medial network) in chronic pain, including structures such as the high medial prefrontal cortex and nucleus accumbens connectivity [10]. Previous studies have shown a correlation between mood disorders, like anxiety and depression, with CPM dysfunction [77]. However, this direction is not well understood: some studies have described an ambivalent relationship, meaning chronic pain leads to depression and anxiety disorders [78]; in contrast, other studies have described the opposite [79,80,81]. Imaging studies report that regions such as the thalamus, insula, ACC, and periaqueductal gray are activated in patients with depression [82,83] and related to EPMS pathways [84]. These structures are also related to learning and memory, suggesting a relationship between chronification of pain and implicit and explicit pain memories; therefore, developing a learning memory of pain might lead to chronic pain. This relationship should be further studied to detangle the relationship between mood disorders and the EPMS healing system.

## 5. Factors That Improve the Balance of the Pain Healing Mechanisms

Several studies have searched for factors that improve the dysfunctional EPMS and pain processing. In this section, we will discuss the most promising interventions.

### 5.1. Exercise

Exercise has shown pain modulation qualities by inducing acute hypoalgesia and pain threshold changes [85]. However, this effect seems to be decreased in chronic pain populations due to the believed relationship between exercise and the activation of the EPMS [86]. Therefore, a disrupted EPMS may be the main explanation for these findings. One hypothesis is that exercise induction hypoalgesia is accomplished by the EPMS system and endorphin system activation, which seems to be associated with muscle nociceptors during physical activity [87]. Similarly, exercise may overstimulate the M1 cortical excitability and induce EPMS activation, leading to better pain outcomes, besides the disruption of the system in chronic pain populations. However, more research into the best exercise modalities and parameters is needed to optimize its use for healing improvement and chronic pain prevention.

### 5.2. Non-Invasive Neuromodulation

Neuromodulation techniques can also enhance the EPMS and thus reduce pain and improve healing. TMS and transcranial direct current stimulation (tDCS) on the primary motor cortex have shown the most promising results in chronic pain populations—TMS seems to be more effective in several neuropathic pain conditions, while tDCS shows positive results on fibromyalgia, spinal cord injury, and low back pain [88,89,90,91]. The mechanistic reasoning behind the stimulation techniques relies on modulating the activity in brain regions involved with pain processing [92]. Imaging studies suggest that motor cortex stimulation reduces pain by modulating the activity in brain networks involved in pain processing, such as the thalamus and the descendent pain modulation system.

Non-invasive brain stimulation techniques are a promising alternative way to modulate the EPMS, trying to restore disrupted pathways and the wholeness of the healing process [93]. The advantages of these techniques include fewer side effects and no risk of addiction compared with common analgesics or opioids, and they can be combined with other behavioral therapies, such as exercise [94,95]. However, tDCS has demonstrated higher applicability due to its safety and portability when compared with TMS. Nowadays, a trend to begin testing tDCS as a home-based intervention [96] could bring higher accessibility to pain treatments focusing on the neurophysiological aspects of the development of chronic pain, restoring the mechanisms of healing. Moreover, since the EPMS activation could also depend on neuro-immune crosstalk from the periphery that is mainly mediated by a vagal feedback loop, the use of vagal nerve stimulation to treat chronic pain is another potential option. There are recent studies showing positive results [97]; however, the literature is still insufficient.

### 5.3. Mind–Body Techniques

The “experience of pain results from what the brain does with sensory input, rather than from the sensory input itself” [98]. Thus, interventions that target the brain circuit and the EPMS could be useful to decrease this type of experience. Mind and body therapies such as hypnosis, meditation, and neurofeedback have shown neuromodulatory effects for reducing pain perception. Hypnosis and hypnotic analgesia have been shown to act in different areas of the cortex and brainstem (anterior cingulate cortex (ACC), primary and secondary somatosensory sensory (S1, S2), bilateral insula, thalamus, pregenual cingulate cortex, pre-supplementary motor area, right prefrontal cortex and striatum, spinothalamic tract), which are related to the EPMS; however, the activation of each area depends on the type of hypnotic suggestion [99].

Meditation practices also provide an analgesic effect; however, its mechanism is not fully understood, and there are still many theories regarding its action [98]. Nonetheless, a randomized cross-over study using mindfulness meditation has shown that it could be related to the recruitment of endogenous opioids during practice that eventually could affect the pain modulation system.

Neurofeedback is a type of biofeedback in which the brain activity of a patient is monitored mainly through an EEG or functional magnetic resonance imaging (fMRI), and the patient can learn how to modify this activity [98]. Different theories explain the rationale behind its effectiveness in pain: a decrease in brain oscillations related to pain or an increase in brain oscillations related to the experience of comfort [98].

These interventions, besides not being completely understood yet, appear as a possible way to modulate the EPMS, including cortex excitability and the descendent pain inhibitory pathways, acting on the healing process.

### 5.4. Pharmacological Neuromodulation

The use of medications is another way to modulate the EPMS. Some types of antidepressants and anticonvulsants have been used in chronic pain treatment. Antidepressant drugs target the non-opioid pain modulation led by serotonin and norepinephrine [100] and are also related to cortical excitability changes, while anticonvulsants act directly on the modulation of cortical excitability.

There are three main reasons to try to recover the endogenous modulation system with antidepressants: (1) the bi-directional relationship between chronic pain and psychiatric disorders, such as depression; (2) their impact on sleep disturbance; and (3) their direct impact on pain relief [101].

The use of anticonvulsants is appropriate mainly for neuropathic pain syndromes. Previous research has shown the impact of these drugs on cortical excitability by multiple mechanisms, including the blocking of sodium channels and calcium channels, the enhancement of inhibitory GABAergic, and the inhibition of glutamatergic neurotransmission [102]. Additionally, some specific types such as pregabalin seem to influence the descendent pain modulation pathways [103]. Consequently, these classes of drugs may act in both mechanisms of healing discussed in this paper.

## 6. Conclusions

In this manuscript, we aimed to raise the hypothesis that the impairment of the EPMS—indexed as an altered cortical excitability and descendent pain modulation pathways—seems to be related to the disruption of the pain healing process and the development of chronic pain. Commonly used biomarkers in the field could be applied to measure the efficiency of the EPMS, such as CPM and motor cortex excitability measured by paired-pulse TMS or EEG. These biomarkers are related to measuring the efficiency of the descendent pain modulation pathway and its associated cortical control (mainly M1 and prefrontal) involved in the activation of the EPMS during and after injury. Chronic pain, such as a variety of neuropsychiatric disorders, has been neglected in the last few years, and many researchers and clinicians lack understanding of the topic. Moreover, in the literature, there is a lack of evidence about chronic pain risk factors, diagnosis, categorization, and treatment. As stated previously, it is a leading cause of years lost disability and costs billions to the government. Future research with further longitudinal studies analyzing the relationship between the main chronic pain biomarkers and its development is necessary. Studies must associate multimodal, dynamic, and simultaneous measurements to better understand the physiopathology of chronic pain and its relationship with the pain sensation and healing process, therefore better understanding the different phenotypes of chronic pain and how to treat them. The acquisition of neurophysiological knowledge of how the chronicity of pain occurs and the EPMS involvement in this process may allow better management of these patients, focusing on chronic pain prevention and the recovery of pain by modulating the EPMS. Investments should be applied to gather data on the main risk factors, diagnostic methods, and different treatment approaches to better identify, prevent, categorize, and, when necessary, treat different kinds of chronic pain patients, improving quality of life and reducing clinical pain.
